# *N*-(2-Meth­oxy­phen­yl)-4-methyl­benzene­sulfonamide

**DOI:** 10.1107/S1600536810042984

**Published:** 2010-10-30

**Authors:** Islam Ullah Khan, Tahir Ali Sheikh, Muhammad Nadeem Arshad

**Affiliations:** a Materials Chemistry Laboratory, Department of Chemistry, GC University, Lahore 54000, Pakistan

## Abstract

In the title compound, C_14_H_15_NO_3_S, the geometry around the S atom of the SO_2_ group is distorted tetra­hedral. The meth­oxy- and methyl-substituted aromatic rings are oriented at a dihedral angle of 71.39 (9)°. Inter­molecular N—H⋯O hydrogen bonds form inversion dimers, which stabilize the crystal structure.

## Related literature

For the anti­microbial activity of sulfonamide compounds, see: Gao & Pederson (2005 ▶). For a related thia­zine mol­ecule, see: Arshad *et al.* (2010 ▶). For a related structure, see: Aziz-ur-Rehman *et al.* (2010 ▶). For graph-set notation, see: Bernstein *et al.* (1995 ▶).
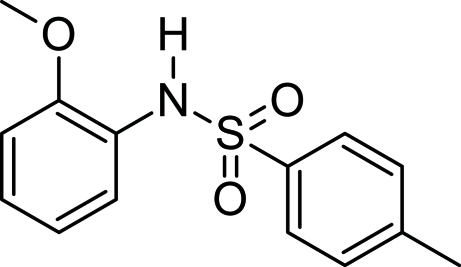


## Experimental

### 

#### Crystal data

C_14_H_15_NO_3_S
*M* *_r_* = 277.33
Orthorhombic, 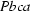

*a* = 12.7395 (9) Å
*b* = 11.4906 (6) Å
*c* = 18.6968 (10) Å
*V* = 2736.9 (3) Å^3^
*Z* = 8
Mo *K*α radiation
μ = 0.24 mm^−1^
*T* = 296 K
0.42 × 0.33 × 0.21 mm

#### Data collection

Bruker Kappa APEXII CCD diffractometer
Absorption correction: multi-scan (*SADABS*; Bruker, 2007 ▶) *T*_min_ = 0.906, *T*_max_ = 0.951
13798 measured reflections
3376 independent reflections
1313 reflections with *I* > 2σ(*I*)
*R*_int_ = 0.086

#### Refinement

*R*[*F* ^2^ > 2σ(*F* ^2^)] = 0.050
*wR*(*F* ^2^) = 0.143
*S* = 0.91
3376 reflections
174 parameters
H-atom parameters constrained
Δρ_max_ = 0.18 e Å^−3^
Δρ_min_ = −0.28 e Å^−3^

### 

Data collection: *APEX2* (Bruker, 2007 ▶); cell refinement: *SAINT* (Bruker, 2007 ▶); data reduction: *SAINT*; program(s) used to solve structure: *SHELXS97* (Sheldrick, 2008 ▶); program(s) used to refine structure: *SHELXL97* (Sheldrick, 2008 ▶); molecular graphics: *ORTEP-3 for Windows* (Farrugia, 1997 ▶) and *PLATON* (Spek, 2009 ▶); software used to prepare material for publication: *WinGX* (Farrugia, 1999 ▶) and *PLATON*.

## Supplementary Material

Crystal structure: contains datablocks I, global. DOI: 10.1107/S1600536810042984/sj5047sup1.cif

Structure factors: contains datablocks I. DOI: 10.1107/S1600536810042984/sj5047Isup2.hkl

Additional supplementary materials:  crystallographic information; 3D view; checkCIF report

## Figures and Tables

**Table 1:** Hydrogen-bond geometry (Å, °)

*D*—H⋯*A*	*D*—H	H⋯*A*	*D*⋯*A*	*D*—H⋯*A*
N1—H1*N*⋯O2^i^	0.84	2.35	3.112 (3)	151

Symmetry code: (i) 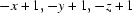.

**Fig. 1.**
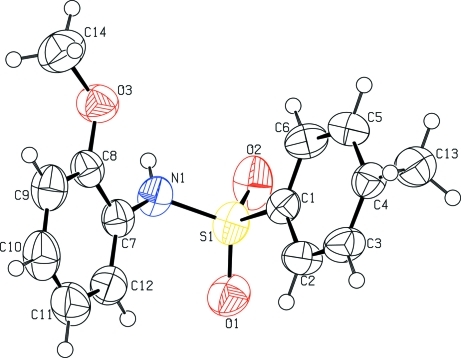
The molecular structure of (I) showing 50% displacement ellipsoids.

**Fig. 2.**
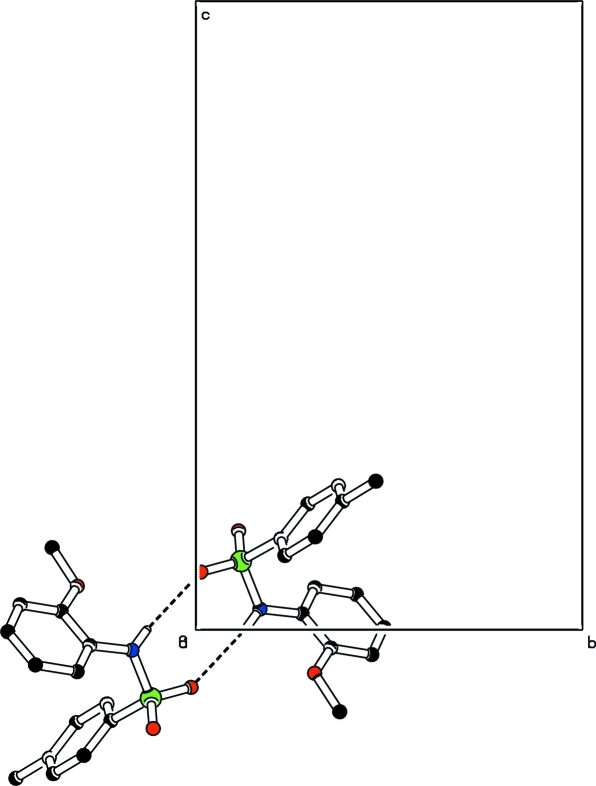
Inversion dimers formed through N—H···O hydrogen bonds drawn as dashed lines.

## supplementary crystallographic information

### Comment

Sulfonamide compounds are well known as antimicrobial agents (Gao & Pederson, 2005). The structure reported here is a precursor used in the synthesis of thiazine heterocycles (Arshad *et al.*, 2010).

The bond lengths and angles in the title compound are similar to those observed in the recently published *N*-(2-methoxyphenyl)benzenesulfonamide (II) (Aziz-ur-Rehman *et al.*, 2010).  The two aromatic rings (C1/C2/C3/C4/C5/C6) and C7/C8/C9/C10/C11/C12) are oriented at dihedral angles of 71.39 (0.09)° unlike the dihedral angles observed for the two independent molecules in II. Similarly the torsion angle C—S—N(H)—C is -56.5 (3) compared with 67.25 (15)° in molecule A and -81.17 (16)° in molecule B of (II). Inversion related intermolecular N—H···O hydrogen bonds form dimers and generate an eight-membered *R*_2_^2^(8) ring motif (Bernstein *et al.*, 1995) Table. 1, Fig. 2.

### Experimental

A mixture of *para* toluenesulfonyl chloride (0.00104 mol; 0.200 g), *o*-anisidine (0.00104 mol; 0.128 g), was stirred in 10–15 ml of distilled water, while maintaining pH of the reaction mixture at 8–10 using 3% sodium carbonate. The progress of the reaction was checked by TLC. On completion of reaction the precipitates obtained were filtered, washed with water and finally dried. Suitable crystals for X-Ray analysis were grown from DCM (dichloromethane) by slow evaporation.

### Refinement

All the C—H and H-atoms were positioned with idealized geometry with C—H = 0.93 Å for aromatic C—H = 0.96 Å and were refined using a riding model with *U*_iso_(H) = 1.2 *U*_eq_(C) for aromatic, with *U*_iso_(H) = 1.5 *U*_eq_(C) for methyl. The N–H H atom was fixed in its found position with *U*_iso_(H) = 1.2 *U*_eq_(N).

### Crystal data

**Table:**

C_14_H_15_NO_3_S	*F*(000) = 1168
*M**_r_* = 277.33	*D*_x_ = 1.346 Mg m^−^^3^
Orthorhombic, *P**b**c**a*	Mo *K*α radiation, λ = 0.71073 Å
Hall symbol: -P 2ac 2ab	Cell parameters from 1013 reflections
*a* = 12.7395 (9) Å	θ = 3.2–19.2°
*b* = 11.4906 (6) Å	µ = 0.24 mm^−^^1^
*c* = 18.6968 (10) Å	*T* = 296 K
*V* = 2736.9 (3) Å^3^	Needle, light brown
*Z* = 8	0.42 × 0.33 × 0.21 mm

### Data collection

**Table:**

Bruker Kappa APEXII CCD diffractometer	3376 independent reflections
Radiation source: fine-focus sealed tube	1313 reflections with *I* > 2σ(*I*)
graphite	*R*_int_ = 0.086
φ and ω scans	θ_max_ = 28.3°, θ_min_ = 3.2°
Absorption correction: multi-scan (*SADABS*; Bruker, 2007)	*h* = −16→10
*T*_min_ = 0.906, *T*_max_ = 0.951	*k* = −11→15
13798 measured reflections	*l* = −24→23

### Refinement

**Table:**

Refinement on *F*^2^	Primary atom site location: structure-invariant direct methods
Least-squares matrix: full	Secondary atom site location: difference Fourier map
*R*[*F*^2^ > 2σ(*F*^2^)] = 0.050	Hydrogen site location: inferred from neighbouring sites
*wR*(*F*^2^) = 0.143	H-atom parameters constrained
*S* = 0.91	*w* = 1/[σ^2^(*F*_o_^2^) + (0.0552*P*)^2^] where *P* = (*F*_o_^2^ + 2*F*_c_^2^)/3
3376 reflections	(Δ/σ)_max_ < 0.001
174 parameters	Δρ_max_ = 0.18 e Å^−^^3^
0 restraints	Δρ_min_ = −0.28 e Å^−^^3^

### Special details

**Table:**

Geometry. All s.u.'s (except the s.u. in the dihedral angle between two l.s. planes) are estimated using the full covariance matrix. The cell s.u.'s are taken into account individually in the estimation of s.u.'s in distances, angles and torsion angles; correlations between s.u.'s in cell parameters are only used when they are defined by crystal symmetry. An approximate (isotropic) treatment of cell s.u.'s is used for estimating s.u.'s involving l.s. planes.
Refinement. Refinement of *F*^2^ against ALL reflections. The weighted *R*-factor *wR* and goodness of fit *S* are based on *F*^2^, conventional *R*-factors *R* are based on *F*, with *F* set to zero for negative *F*^2^. The threshold expression of *F*^2^ > 2σ(*F*^2^) is used only for calculating *R*-factors(gt) *etc*. and is not relevant to the choice of reflections for refinement. *R*-factors based on *F*^2^ are statistically about twice as large as those based on *F*, and *R*- factors based on ALL data will be even larger.

### Fractional atomic coordinates and isotropic or equivalent isotropic displacement parameters (Å^2^)

**Table:**

	*x*	*y*	*z*	*U*_iso_*/*U*_eq_	
S1	0.51740 (7)	0.61649 (6)	0.39096 (4)	0.0571 (3)	
O1	0.60405 (18)	0.61039 (18)	0.34278 (11)	0.0706 (7)	
N1	0.55946 (19)	0.6656 (2)	0.46723 (12)	0.0529 (7)	
C1	0.4303 (2)	0.7199 (2)	0.35541 (14)	0.0465 (7)	
O2	0.46120 (19)	0.51265 (17)	0.40827 (11)	0.0751 (7)	
C2	0.4619 (3)	0.7899 (3)	0.30004 (16)	0.0584 (8)	
H2	0.5298	0.7842	0.2822	0.070*	
O3	0.49484 (18)	0.80720 (18)	0.56921 (11)	0.0662 (6)	
C3	0.3924 (3)	0.8688 (3)	0.27103 (16)	0.0607 (9)	
H3	0.4146	0.9162	0.2337	0.073*	
C4	0.2908 (3)	0.8796 (2)	0.29578 (15)	0.0510 (8)	
C5	0.2613 (3)	0.8091 (3)	0.35213 (17)	0.0598 (9)	
H5	0.1935	0.8150	0.3703	0.072*	
H1N	0.5306	0.6270	0.4999	0.072*	
C6	0.3298 (3)	0.7303 (3)	0.38188 (16)	0.0610 (9)	
H6	0.3082	0.6839	0.4199	0.073*	
C7	0.6101 (2)	0.7748 (2)	0.47481 (15)	0.0481 (8)	
C8	0.5756 (2)	0.8488 (2)	0.52917 (16)	0.0491 (8)	
C9	0.6239 (3)	0.9550 (3)	0.53963 (18)	0.0650 (9)	
H9	0.6006	1.0047	0.5755	0.078*	
C10	0.7065 (3)	0.9868 (3)	0.4967 (2)	0.0742 (11)	
H10	0.7391	1.0583	0.5038	0.089*	
C11	0.7412 (3)	0.9152 (3)	0.4440 (2)	0.0766 (11)	
H11	0.7969	0.9380	0.4151	0.092*	
C12	0.6934 (3)	0.8076 (3)	0.43331 (16)	0.0629 (9)	
H12	0.7182	0.7579	0.3979	0.075*	
C13	0.2160 (3)	0.9656 (3)	0.26372 (17)	0.0697 (10)	
H13A	0.2137	0.9552	0.2128	0.105*	
H13B	0.2391	1.0432	0.2745	0.105*	
H13C	0.1472	0.9536	0.2833	0.105*	
C14	0.4637 (3)	0.8720 (3)	0.63051 (18)	0.0889 (12)	
H14A	0.4396	0.9476	0.6159	0.133*	
H14B	0.5224	0.8804	0.6623	0.133*	
H14C	0.4079	0.8318	0.6547	0.133*	

### Atomic displacement parameters (Å^2^)

**Table:**

	*U*^11^	*U*^22^	*U*^33^	*U*^12^	*U*^13^	*U*^23^
S1	0.0728 (6)	0.0410 (5)	0.0575 (5)	0.0023 (4)	−0.0082 (5)	−0.0047 (4)
O1	0.0764 (17)	0.0696 (16)	0.0659 (14)	0.0201 (12)	0.0028 (13)	−0.0123 (12)
N1	0.0685 (19)	0.0410 (14)	0.0491 (14)	−0.0108 (13)	−0.0068 (13)	0.0069 (12)
C1	0.057 (2)	0.0393 (17)	0.0436 (16)	−0.0066 (15)	0.0000 (15)	−0.0013 (14)
O2	0.104 (2)	0.0370 (12)	0.0845 (16)	−0.0171 (12)	−0.0261 (14)	0.0047 (11)
C2	0.051 (2)	0.064 (2)	0.0603 (19)	0.0033 (17)	0.0091 (17)	0.0070 (17)
O3	0.0647 (17)	0.0638 (14)	0.0700 (14)	−0.0036 (12)	0.0084 (12)	−0.0086 (12)
C3	0.067 (2)	0.064 (2)	0.0515 (19)	0.0029 (19)	0.0090 (18)	0.0179 (17)
C4	0.056 (2)	0.0500 (19)	0.0469 (18)	0.0010 (16)	−0.0005 (16)	−0.0061 (16)
C5	0.051 (2)	0.063 (2)	0.065 (2)	−0.0016 (17)	0.0114 (17)	0.0007 (18)
C6	0.070 (3)	0.057 (2)	0.0552 (19)	−0.0038 (18)	0.0124 (19)	0.0129 (17)
C7	0.052 (2)	0.0433 (18)	0.0492 (18)	−0.0005 (16)	−0.0084 (16)	0.0078 (15)
C8	0.047 (2)	0.046 (2)	0.0543 (18)	−0.0001 (16)	−0.0065 (16)	0.0053 (15)
C9	0.078 (3)	0.050 (2)	0.066 (2)	−0.007 (2)	−0.014 (2)	−0.0039 (18)
C10	0.084 (3)	0.060 (2)	0.079 (2)	−0.024 (2)	−0.022 (2)	0.010 (2)
C11	0.072 (3)	0.086 (3)	0.072 (3)	−0.028 (2)	−0.007 (2)	0.020 (2)
C12	0.065 (2)	0.069 (2)	0.055 (2)	−0.0093 (19)	−0.0024 (18)	0.0010 (18)
C13	0.068 (3)	0.075 (2)	0.066 (2)	0.015 (2)	0.0018 (19)	0.0041 (18)
C14	0.098 (3)	0.100 (3)	0.069 (2)	0.005 (2)	0.012 (2)	−0.011 (2)

### Geometric parameters (Å, °)

**Table:**

S1—O1	1.426 (2)	C6—H6	0.9300
S1—O2	1.429 (2)	C7—C12	1.368 (4)
S1—N1	1.625 (2)	C7—C8	1.396 (4)
S1—C1	1.756 (3)	C8—C9	1.381 (4)
N1—C7	1.418 (3)	C9—C10	1.373 (5)
N1—H1N	0.8394	C9—H9	0.9300
C1—C2	1.371 (4)	C10—C11	1.357 (5)
C1—C6	1.378 (4)	C10—H10	0.9300
C2—C3	1.378 (4)	C11—C12	1.393 (4)
C2—H2	0.9300	C11—H11	0.9300
O3—C8	1.359 (3)	C12—H12	0.9300
O3—C14	1.424 (3)	C13—H13A	0.9600
C3—C4	1.379 (4)	C13—H13B	0.9600
C3—H3	0.9300	C13—H13C	0.9600
C4—C5	1.382 (4)	C14—H14A	0.9600
C4—C13	1.498 (4)	C14—H14B	0.9600
C5—C6	1.375 (4)	C14—H14C	0.9600
C5—H5	0.9300		
			
O1—S1—O2	119.32 (14)	C12—C7—N1	122.7 (3)
O1—S1—N1	108.43 (13)	C8—C7—N1	117.9 (3)
O2—S1—N1	104.86 (12)	O3—C8—C9	124.8 (3)
O1—S1—C1	106.45 (14)	O3—C8—C7	115.1 (3)
O2—S1—C1	109.52 (15)	C9—C8—C7	120.1 (3)
N1—S1—C1	107.79 (13)	C10—C9—C8	119.6 (3)
C7—N1—S1	123.03 (19)	C10—C9—H9	120.2
C7—N1—H1N	126.5	C8—C9—H9	120.2
S1—N1—H1N	108.1	C11—C10—C9	120.9 (3)
C2—C1—C6	119.5 (3)	C11—C10—H10	119.6
C2—C1—S1	119.8 (3)	C9—C10—H10	119.6
C6—C1—S1	120.6 (2)	C10—C11—C12	120.0 (4)
C1—C2—C3	119.7 (3)	C10—C11—H11	120.0
C1—C2—H2	120.2	C12—C11—H11	120.0
C3—C2—H2	120.2	C7—C12—C11	120.1 (3)
C8—O3—C14	118.1 (3)	C7—C12—H12	119.9
C2—C3—C4	122.0 (3)	C11—C12—H12	119.9
C2—C3—H3	119.0	C4—C13—H13A	109.5
C4—C3—H3	119.0	C4—C13—H13B	109.5
C3—C4—C5	117.3 (3)	H13A—C13—H13B	109.5
C3—C4—C13	121.4 (3)	C4—C13—H13C	109.5
C5—C4—C13	121.2 (3)	H13A—C13—H13C	109.5
C6—C5—C4	121.4 (3)	H13B—C13—H13C	109.5
C6—C5—H5	119.3	O3—C14—H14A	109.5
C4—C5—H5	119.3	O3—C14—H14B	109.5
C5—C6—C1	120.1 (3)	H14A—C14—H14B	109.5
C5—C6—H6	120.0	O3—C14—H14C	109.5
C1—C6—H6	120.0	H14A—C14—H14C	109.5
C12—C7—C8	119.3 (3)	H14B—C14—H14C	109.5
			
O1—S1—N1—C7	58.3 (3)	C2—C1—C6—C5	1.0 (5)
O2—S1—N1—C7	−173.2 (2)	S1—C1—C6—C5	−178.0 (2)
C1—S1—N1—C7	−56.5 (3)	S1—N1—C7—C12	−51.4 (4)
O1—S1—C1—C2	−11.4 (3)	S1—N1—C7—C8	131.6 (2)
O2—S1—C1—C2	−141.7 (2)	C14—O3—C8—C9	−6.2 (4)
N1—S1—C1—C2	104.7 (2)	C14—O3—C8—C7	173.0 (3)
O1—S1—C1—C6	167.6 (2)	C12—C7—C8—O3	−177.7 (3)
O2—S1—C1—C6	37.3 (3)	N1—C7—C8—O3	−0.6 (4)
N1—S1—C1—C6	−76.3 (3)	C12—C7—C8—C9	1.5 (4)
C6—C1—C2—C3	−0.7 (4)	N1—C7—C8—C9	178.6 (3)
S1—C1—C2—C3	178.3 (2)	O3—C8—C9—C10	178.5 (3)
C1—C2—C3—C4	−0.3 (5)	C7—C8—C9—C10	−0.6 (5)
C2—C3—C4—C5	1.0 (5)	C8—C9—C10—C11	0.1 (5)
C2—C3—C4—C13	−179.9 (3)	C9—C10—C11—C12	−0.5 (6)
C3—C4—C5—C6	−0.7 (5)	C8—C7—C12—C11	−1.8 (5)
C13—C4—C5—C6	−179.8 (3)	N1—C7—C12—C11	−178.9 (3)
C4—C5—C6—C1	−0.3 (5)	C10—C11—C12—C7	1.3 (5)

### Hydrogen-bond geometry (Å, °)

**Table:**

*D*—H···*A*	*D*—H	H···*A*	*D*···*A*	*D*—H···*A*
N1—H1N···O2^i^	0.84	2.35	3.112 (3)	151

Symmetry codes: (i) −*x*+1, −*y*+1, −*z*+1.
